# Use of Nanopore Sequencing to Characterise the Genomic Architecture of Mobile Genetic Elements Encoding *bla*_CTX-M-15_ in *Escherichia coli* Causing Travellers’ Diarrhoea

**DOI:** 10.3389/fmicb.2022.862234

**Published:** 2022-03-29

**Authors:** Matthew T. Bird, David R. Greig, Satheesh Nair, Claire Jenkins, Gauri Godbole, Saheer E. Gharbia

**Affiliations:** ^1^National Infection Service, UK Health Security Agency, London, United Kingdom; ^2^Health Protection Research Unit in Genomes and Enabling Data, Warwick, United Kingdom; ^3^NIRH Health Protection Research Unit for Gastrointestinal Pathogens, Liverpool, United Kingdom; ^4^Division of Infection and Immunity, The Roslin Institute and Royal (Dick) School of Veterinary Studies, University of Edinburgh, Edinburgh, United Kingdom

**Keywords:** *bla*_CTX-M-15_, chromosomal integration, nanopore sequencing, antibiotic resistance, mobile genetic element, plasmid

## Abstract

Increasing levels of antimicrobial resistance (AMR) have been documented in *Escherichia coli* causing travellers’ diarrhoea, particularly to the third-generation cephalosporins. Diarrhoeagenic *E. coli* (DEC) can act as a reservoir for the exchange of AMR genes between bacteria residing in the human gut, enabling them to survive and flourish through the selective pressures of antibiotic treatments. Using Oxford Nanopore Technology (ONT), we sequenced eight isolates of DEC from four patients’ specimens who had all recently returned to the United Kingdome from Pakistan. Sequencing yielded two DEC harbouring *bla*_CTX-M-15_ per patient, all with different sequence types (ST) and belonging to five different pathotypes. The study aimed to determine whether *bla*_CTX-M-15_ was located on the chromosome or plasmid and to characterise the drug-resistant regions to better understand the mechanisms of onward transmission of AMR determinants. Patients A and C both had one isolate where *bla*_CTX-M-15_ was located on the plasmid (899037 & 623213, respectively) and one chromosomally encoded (899091 & 623214, respectively). In patient B, *bla*_CTX-M-15_ was plasmid-encoded in both DEC isolates (786605 & 7883090), whereas in patient D, *bla*_CTX-M-15_ was located on the chromosome in both DEC isolates (542093 & 542099). The two *bla*_CTX-M-15_-encoding plasmids associated with patient B were different although the *bla*_CTX-M-15_-encoding plasmid isolated from 788309 (IncFIB) exhibited high nucleotide similarity to the *bla*_CTX-M-15_-encoding plasmid isolated from 899037 (patient A). In the four isolates where *bla*_CTX-M-15_ was chromosomally encoded, two isolates (899091 & 542099) shared the same insertion site. The *bla*_CTX-M-15_ insertion site in isolate 623214 was described previously, whereas that of isolate 542093 was unique to this study. Analysis of Nanopore sequencing data enables us to characterise the genomic architecture of mobile genetic elements encoding AMR determinants. These data may contribute to a better understanding of persistence and onward transmission of AMR determinants in multidrug-resistant (MDR) *E. coli* causing gastrointestinal and extra-intestinal infections.

## Introduction

In recent years, there has been an increasing level of antimicrobial resistance (AMR) reported on a global scale which threatens the achievements of 21st century modern medicine in its ability to treat and prevent common bacterial infections. AMR is a global problem but disproportionally affects certain regions such as the Indian sub-continent, Africa and Latin America (Shawa et al., 2021). The prevalence of AMR is high in Lower Middle-Income Countries (LMIC) due to excessive use of antibiotics in both clinical and agricultural settings, high population density and low levels of sanitation (Bevan et al., 2018; Mazumder et al., 2020). Travellers to high-risk countries may become colonised with AMR bacterial strains within their gut microbiome potentially resulting in horizontal transfer events, persistence and spread of AMR determinants within their respective home country (Bevan et al., 2021). Therefore, routine surveillance of AMR is crucial to better understand the mechanisms of transmission and to limit the threat to public health.

AMR can be conferred by chromosomal mutations in housekeeping genes or by the acquisition of mobile genetic elements (MGE) encoding a wide variety of AMR determinants that may or may not be incorporated into the chromosome. The majority of gastrointestinal (GI) infections is mild and self-limiting and therefore does not require antimicrobial treatment. However, antibiotics may be required if the patient is very old, very young or immunocompromised (Casburn-Jones and Farthing, 2004; Guarino et al., 2018). Therefore, monitoring AMR in GI pathogens is essential to inform treatment guidelines. Furthermore, GI bacterial pathogens can act as a reservoir for AMR determinants in the gut of humans and animals (Minja et al., 2021). The gut microbiome facilitates the transfer of AMR determinants mainly *via* horizontal gene transfer (HGT) due to its high density and diversity of bacterial microorganisms (Penders et al., 2013). The study of GI pathogens can provide insight into the source persistence and transmissibility of AMR determinants in the gut.

One of the most extensively studied GI bacteria, *Escherichia coli*, can live harmlessly in the gut microbiome but can become pathogenic through the acquisition of virulence factors, such as genes encoding adherence mechanisms, invasions and toxins. *E. coli* are promiscuous and HGT between bacteria is common in the gut environment (Huang et al., 2017). Diarrhoeagenic *Escherichia coli* (DEC) are *E. coli* strains that cause diarrheal disease and comprise several distinct pathotypes enteropathogenic *E. coli* (EPEC), enterohemorrhagic/Shiga toxin-producing *E. coli* (EHEC/STEC), enteroaggregative *E. coli* (EAEC), enterotoxigenic *E. coli* (ETEC) and enteroinvasive *E. coli* (EIEC; Kaper et al., 2004; Gomes et al., 2016). DEC are a common cause of travellers’ diarrhoea and are prevalent in countries where antibiotic use is poorly regulated, and high levels of multidrug resistance in DEC have been detected (Do Nascimento et al., 2017; Boxall et al., 2020).

ß-lactam antibiotics are a common treatment for bacterial infections in humans and animals. AMR surveillance, by whole-genome sequencing (WGS), has seen a prominent rise in resistance to this class of antibiotics with specific proliferation of variants in the *bla*_CTX-M_ group of enzymes belonging to extended-spectrum β-lactamases (ESBLs; Shaikh et al., 2015). Processes such as horizontal gene transfer and especially conjugation have driven the emergence and dissemination of ESBLs in *E. coli* as the enzymes are usually encoded on plasmids (Irrgang et al., 2017). The monitoring of this spread of ESBL clones is crucial due to their capability to hydrolyse third-generation cephalosporins of which there is currently no safe or effective treatment options available for carbapenem-resistant pathogens (Agyekum et al., 2016; Gekenidis et al., 2020). Due to this increased prevalence of *bla*_CTX-M_ type enzymes, identification of plasmids encoding *bla*_CTX-M_ derivatives is becoming more frequent (Shawa et al., 2021). Furthermore, due to plasmids highly transmissible nature, the incorporation of *bla*_CTX-M_ derivatives into the chromosome is becoming common and facilitates the persistence and spread of AMR clones (Irrgang et al., 2017; Lee et al., 2021).

Short read sequencing is still widely utilised for AMR detection, but *E. coli* genomes generated in this way are characteristically challenging to assemble *de novo* (Wick et al., 2017). In contrast, long-read sequencing can provide the data and the resolution to support the construction of closed chromosomal and plasmid genomes, enabling us to study the genetic environment of each AMR determinant (Loman et al., 2015).

Using Oxford Nanopore Technology (ONT), we sequenced four pairs of *E. coli* isolates (each pair from a single patient) with eight different sequence types (ST) belonging to five different pathotypes of extended-ß-lactamase-producing DEC harbouring *bla*_CTX-M-15_ from four patients recently returned to the United Kingdome from Pakistan. The study aimed to determine whether *bla*_CTX-M-15_ was chromosome or plasmid-encoded and to characterise the drug-resistant region to better understand the mechanisms of onward transmission of AMR determinants.

## Materials and Methods

### Data Collection and Bacterial Strains

Faecal specimens from patients with symptoms of gastrointestinal disease that test negative for *Salmonella*, *Shigella* and *Campylobacter* species and *E. coli* O157 at the local hospital laboratory can be submitted to the Gastrointestinal Bacterial Reference Unit (GBRU) at United Kingdome Health Security Agency (UKHSA) for further investigation. This includes testing for DEC pathotypes by PCR and subsequent culture. WGS of all isolates was implemented in July 2015 as part of routine surveillance.

In this study, faecal specimens were selected from four patients (two male and two female) who were each infected with two pathotypes of DEC exhibiting resistance to the third-generation cephalosporins. Patients A and D were both female (aged 24 and 79, respectively) and had both travelled to Pakistan in 2019. On the other hand, patients B and C were both male (aged 6 and 7, respectively) but had both travelled to Pakistan in 2018 (Table 1). Illumina sequencing was performed on a single colony of each DEC pathotype from each faecal specimen on the date; the colonies were isolated at UKHSA. The same single colony that was sequenced on the Illumina platform was stored in the UKHSA archive and revived for Nanopore sequencing 12 months later.

**Table 1:** Summary of patient data including Pathotype, Serotype and Sequence type.

Patient	A		B		C		D	
Isolate	899091	899037	786605	788309	623214	623213	542093	542099
Pathotype	EIEC	ETEC	STEC	EAEC	EAEC	ETEC	EAEC	EPEC
Serotype	O96:H19	O167:H5	O117:H7	O:H31	O51:H30	O167:H41	O:H21	O142:H6
Sequence Type	ST99	ST443	ST504	ST3032	ST38	ST182	ST227	ST1283
*bla*_CTX-M-15_ position	Chromosome	Plasmid	Plasmid	Plasmid	Chromosome	Plasmid	Chromosome	Chromosome
Plasmid inc type	N/A	IncFIB	IncI1	IncFIB	N/A	IncX1	N/A	N/A
Date of travel	08/2019		07/2018		10/2018		05/2019	
Gender	Female		Male		Male		Female	
Age	80		24		8		6	

*EIEC, Enteroinvasive E. coli; ETEC, Enterotoxigenic E. coli; STEC, Shiga toxin-producing E. coli; EAEC, Enteroaggregative E. coli; and EPEC, Enteropathogenic E. coli. Bla_CTX-M-15_ position row indicates whether bla_CTX-M-15_ was encoded on the chromosome or plasmid*.

### Short-Read Sequencing on the Illumina HiSeq 2500

Illumina sequencing was performed at UKHSA and followed the same protocol as described by Chattaway et al. (2017). The QIAsymphony system (Qiagen) was used to extract genomic DNA from selected DEC samples. The Nextera XP kit (Illumina) was used to prepare the sequence library for sequencing on the HiSeq 2,500 instrument (Illumina), run with the fast protocol. Trimmomatic v0.27 was utilised to remove bases with a PHRED score of <30 from the leading and trailing ends on the FASTQ reads, with reads <50 bp after quality trimming discarded (Bolger et al., 2014).

**Figure 1:**
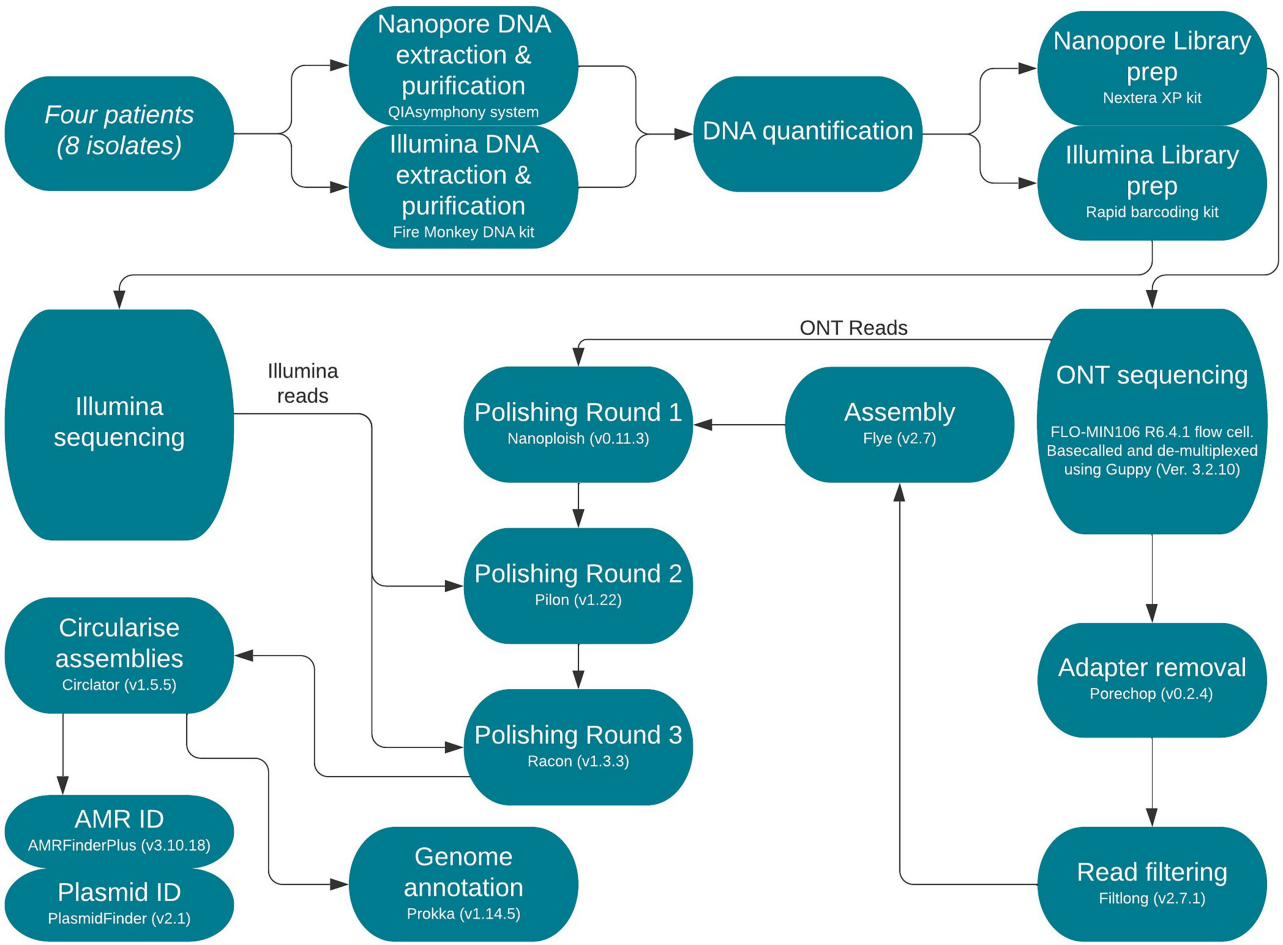
Flow diagram of end-to-end protocol. Version numbers of programmes used are included and were necessary sequencing kits and read types are annotated.

Sequence type (ST) was determined from reads using MOST (v1.0) as previously described by Tewolde et al. (2016) and eBurst Group (eBG) as described in Achtman et al. (2012; Figure 1).

### Nanopore Sequencing Using ONT and Data Processing

Fire Monkey DNA extraction kit (Revolugen) was used to extract and purify high-molecular-weight genomic DNA following the manufacturer’s instructions. Qubit and the HS (high sensitivity) dsDNA assay kit (Thermo fisher Scientific) was then used to quantify genomic DNA for each extract, following the manufacturer’s instructions as described by Yara et al. (2020; Figure 1).

The Rapid barcoding kit SQK-RBK004 (Oxford Nanopore Technologies) was used for library preparation. The prepared libraries were loaded into a FLO-MIN106 R9.4.1D flow cell (Oxford Nanopore Technologies) and sequenced using the MinION (Oxford Nanopore Technologies) for 48 h. Data produced in a raw FAST5 format were base called and de-multiplexed using Guppy v3.2.10 (Oxford Nanopore Technologies) using the FAST protocol (Oxford Nanopore Technologies) into FASTQ format and de-multiplexed into each samples’ respective barcode.

Nanopore reads were trimmed and filtered using Porechop v0.2.4^1^ and Filtlong v0.2.0,^2^ respectively, as previously described (Greig et al., 2021).

### *De novo* Assembly, Polishing and Annotation

Trimmed and filtered Nanopore FASTQ files were assembled using Flye v2.7, with the following parameter, min-overlap = 4,000 (minimum overlap between reads; Kolmogorov et al., 2019). Correction of the draft assembly occurred in a three-step process using Nanopolish v0.11.3 (Loman et al., 2015) using Nanopore reads followed by Pilon v1.4.3 (Walker et al., 2014) using Illumina reads for each sample and finally, Racon v1.4.3 (Vaser et al., 2017) also using Illumina reads as previously described (Greig et al., 2021). Prokka v1.14.5 (Seemann, 2014) was applied to annotate the genomes (Figure 1).

### Antimicrobial Resistance Gene Identification and Plasmid Typing

*In silico* identification of AMR genes among the isolates in this study was performed using AMRFinderPlus v3.10 (Feldgarden et al., 2021). Manual review of AMR results was conducted in Artemis v18.1.0 (Carver et al., 2012) to ensure correct identification and whether *bla*_CTX-M-15_ was encoded on the chromosome or a resistance plasmid. PlasmidFinder v2.1^3^ was utilised to identify any plasmid replicons (Figure 1).

### NCBI BLAST

The National Centre for Biotechnology Information (NCBI) BLAST (Rédei, 2008) database was utilised to identify publicly available plasmids encoding or chromosomal integrations of *bla*_CTX-M-15_ that shared significant nucleotide similarity to the plasmids and chromosomal integrations we outlined in this study. BLAST was used over other complied databases such as PLSDB (Galata et al., 2019) due to BLAST having chromosomal data which could help us identify if any of our plasmids had been integrated into the chromosome. PLSDB was used to verify consistency between best hits identified on BLAST (Supplementary Table 7).

Chromosomal regions containing *bla*_CTX-M-15_ had higher sequence similarity to publicly available plasmids encoding *bla*_CTX-M-15_ or other chromosomal integrations of *bla*_CTX-M-15_. The NCBI BLAST database was also used to find similar publicly available *bla*_CTX-M-15_-encoding plasmids to compare to the *bla*_CTX-M-15_-encoding plasmids identified in this study.

### Visualisation Tools

Genome level alignments were made in Mauve v2.4.0 (Darling et al., 2004). Chromosomal integration comparisons were made using Artemis v18.1.0 and EasyFig v2.2.5, respectively (Sullivan et al., 2011; Carver et al., 2012). Plasmid comparisons were conducted using BRIG v0.95 (Alikhan et al., 2011; Figure 1).

### Data Deposition

Illumina and Nanopore FASTQ files are available from NCBI BioProject PRJNA315192. The SRA (sequence read archive) accession numbers for both technologies are in Supplementary Table 1. The outbreak sample finalised assemblies can also be found under BioProject PRJNA315192 and the GenBank accession numbers are located in Supplementary Table 2.

## Results

### Strain Characteristics

The eight DEC selected for this study were isolated from the faecal specimens of four patients (two isolates per one patient) who all presented with symptoms of traveller’s diarrhoea when returning to the United Kingdom (UK) from Pakistan. Patient A was infected with (899091) EIEC ST99 O96:H19 & (899037) ETEC ST443 O167:H5; Patient B was infected with (786605) STEC ST504 O117:H7 & (788309) EAEC ST3032 O unidentifiable:H31; Patient C was infected with (623214) EAEC ST38 O51:H30 & (623213) ETEC ST182 O167:H41; and Patient D was infected with (542093) EAEC ST227 O unidentifiable:H21 & (542099) EPEC ST1283 O142:H6 (Table 1).

*In silico* antimicrobial analysis, profiles revealed a total of 24 AMR genes (belonging to nine classes; Supplementary Tables 3, 4) that confer resistance to antibiotics that are commonly used in a clinical setting. β-lactamase genes were among the most common identified with *bla*_CTX-M-15_ being identified in every sample.

Analysis of the long-read sequencing data revealed that the eight isolates all assembled into one circular chromosome which ranged from 4,854,807 bp (542099) to 5,323,098 bp (623214) and contained two to four different plasmids (Supplementary Table 5). The *bla*_CTX-M-15_ gene was encoded on a plasmid in four isolates (899037, 786605, 788309 & 623213), but in the remaining four isolates (899091, 623214, 542093 & 542099), it was encoded on the chromosome (Table 1). Manual review of these results was conducted in Artemis v18.1.0 (Carver et al., 2012) to ensure correct identification and whether *bla*_CTX-M-15_ was encoded on the chromosome or a resistance plasmid.

### Plasmid Characterisation Using Nanopore Sequencing Data

In patient B, *bla*_CTX-M-15_ was plasmid-encoded in both DEC isolates 786605 (IncI1) & 788309 (IncFIB), whereas, in patient D, *bla*_CTX-M-15_ was encoded on the chromosome in both DEC isolates (542093 & 542099). Patients A and C both had one isolate, 899037 (IncFIB) & 623214 (IncX1), where *bla*_CTX-M-15_ was encoded on the plasmid and another isolate where *bla*_CTX-M-15_ was encoded on the chromosome (899091 & 623214, respectively; Table 1).

#### p788309 and p899037

**Figure 2:**
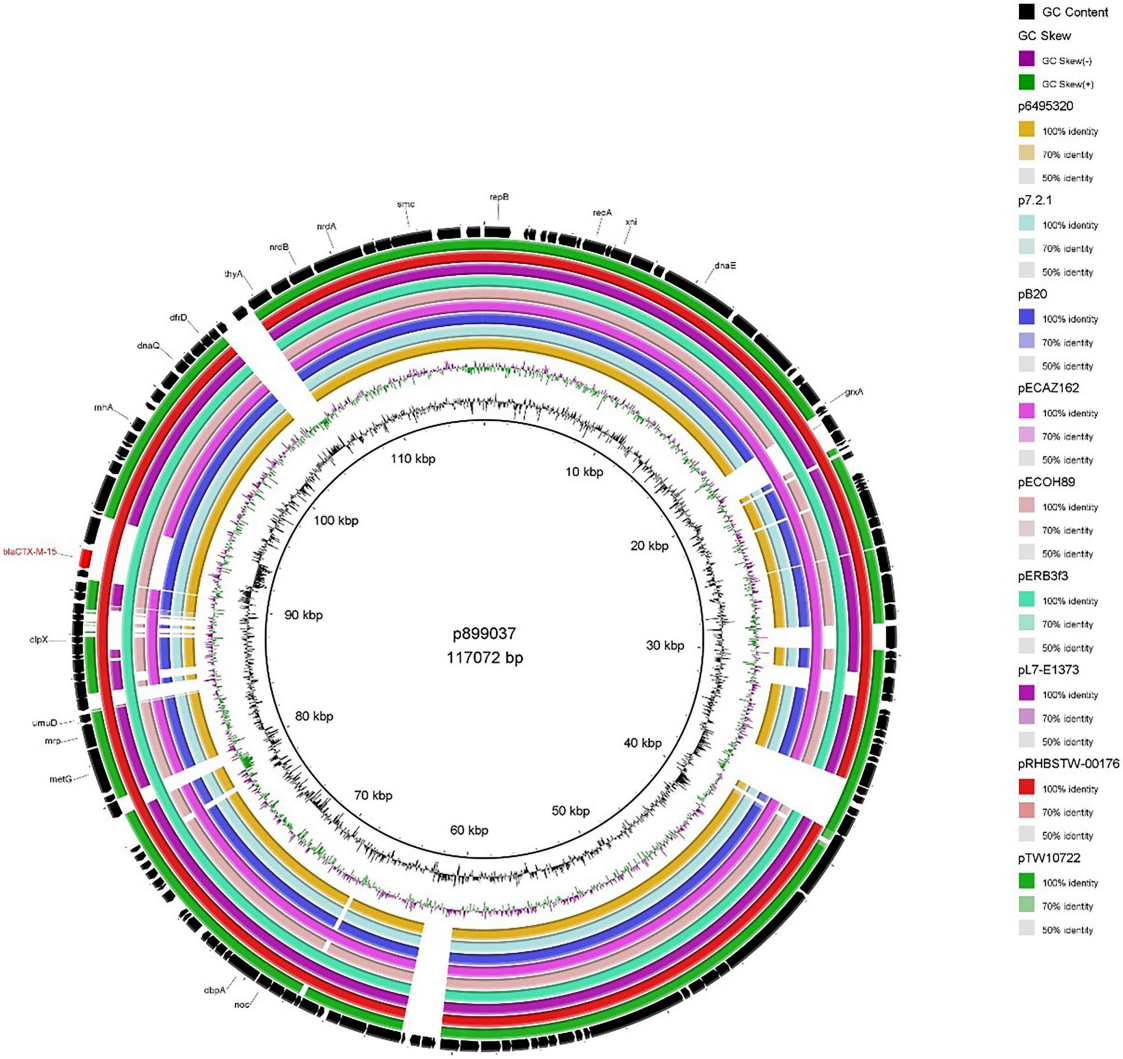
BRIG plot comparing p899037 to nine publicly available *bla*_CTX-M-15_ encoding IncFIB plasmids. AMR genes are coloured red, and all other genes are coloured black. Coloured circles represent isolates with a gradient on similarity.

**Figure 3:**
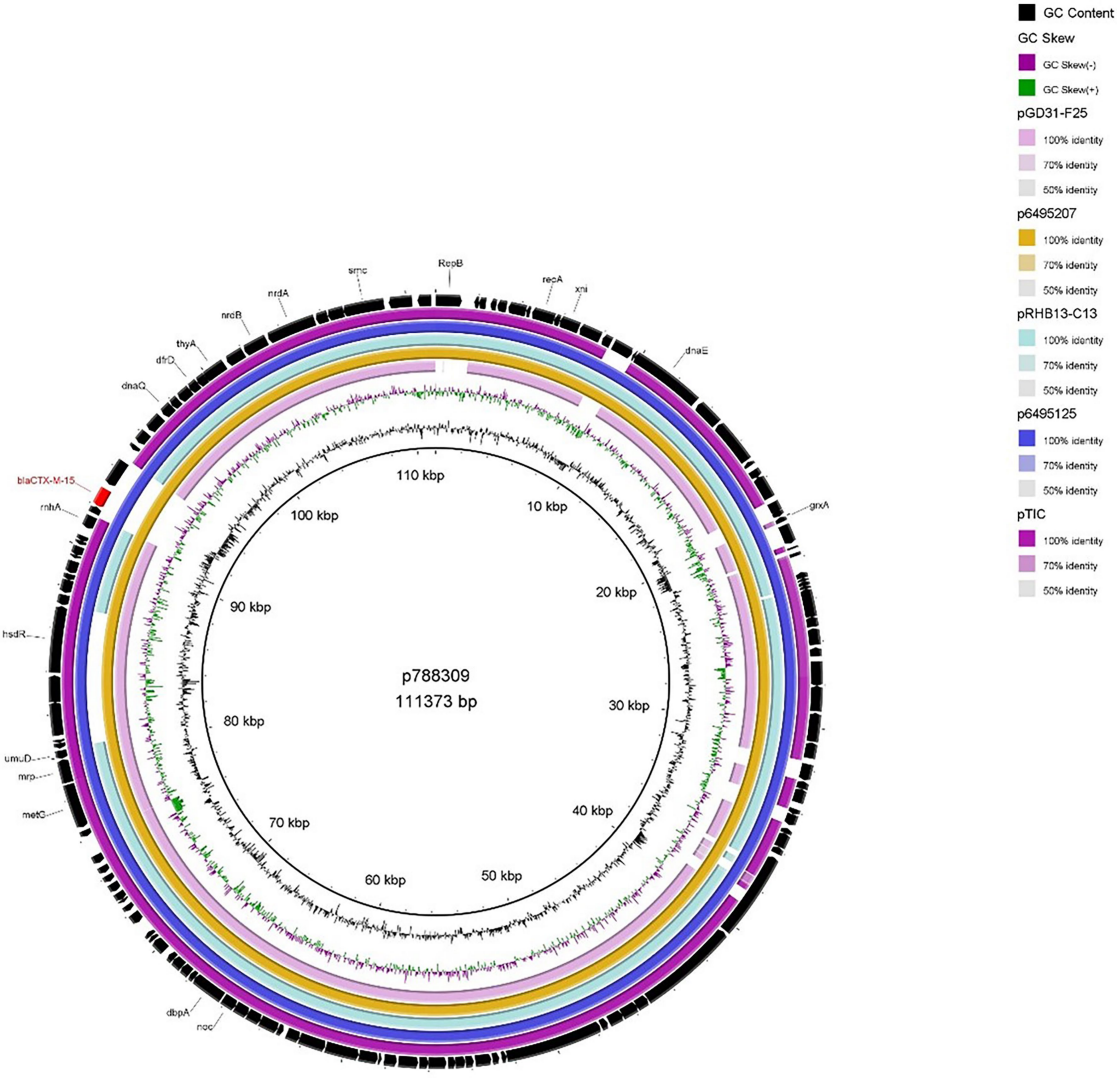
BRIG plot comparing p788309 to five publicly available *bla*_CTX-M-15_ encoding IncFIB plasmids. AMR genes are coloured red, and all other genes are coloured black. Coloured circles represent isolates with a gradient on similarity.

The two IncFIB plasmids (isolated from 899037 & 788309; Figures 2, 3) exhibited 96.98% sequence nucleotide similarity (84% query coverage) despite being associated with two different patients (Patient A & B, respectively) and host cells with different serotypes, pathotypes and sequence types. Further investigation using NCBI BLAST (Rédei, 2008) highlighted that p788309 (Figure 3) showed a high sequence nucleotide sequence identity of 99.99% (100% query coverage) to both p6495207 (accession number: LR595878.1) and p6495125 (accession number: LR595890.1) which were two unnamed *E. coli* hospital plasmids sequenced by the Sanger Institute in 2019. Comparatively p899037 showed a 100% percent identity (94% query coverage) to both pERB3f3 (accession number: MV590712.1) and pRHBSTW-00176 (accession number: CP056801.1; Figure 2). pERB3f3 was associated with a traveller returning to the United Kingdome from India (Bevan et al., 2021), providing further evidence that south Asia is possibly a hotspot for ESBL-producing *E. coli* and highlighting the role human migration and travel plays in the spread of ESBL (& other AMR genes) globally. Furthermore, previous studies showed that over 80% of travellers returning from South Asia became colonised with ESBL-producing *E. coli* (Bevan et al., 2018).

#### p786605

In contrast to the IncFIB plasmids described above, p786605 (IncI1 plasmid) encoded several AMR determinants (*dfrA1, aadA1, bla*_TEM_*, sul2* and *qnrS1*) in addition to *bla*_CTX-M-15_. MDR *bla*_CTX-M-15_-encoding plasmids have been identified in previous literature but are most commonly associated with IncFII-FIA-FIB plasmid replicons (Awosile and Agbaje, 2021; Minja et al., 2021). Analysis also identified the *intI1* gene, suggesting the presence of a class 1 (C1) integron (Ghaly et al., 2017; Awosile and Agbaje, 2021). Comparison to the general structure of C1 integrons outlined by Kubomura et al. (2020) showed p786605 contains a truncated C1 integron encoding *dfrA1*, *aadA1* & *bla*_TEM_. Other AMR determinants—*sul2*, *qnrS1* and *bla*_CTX-M-15_—were encoded close downstream of this truncated C1 integron.

**Figure 4:**
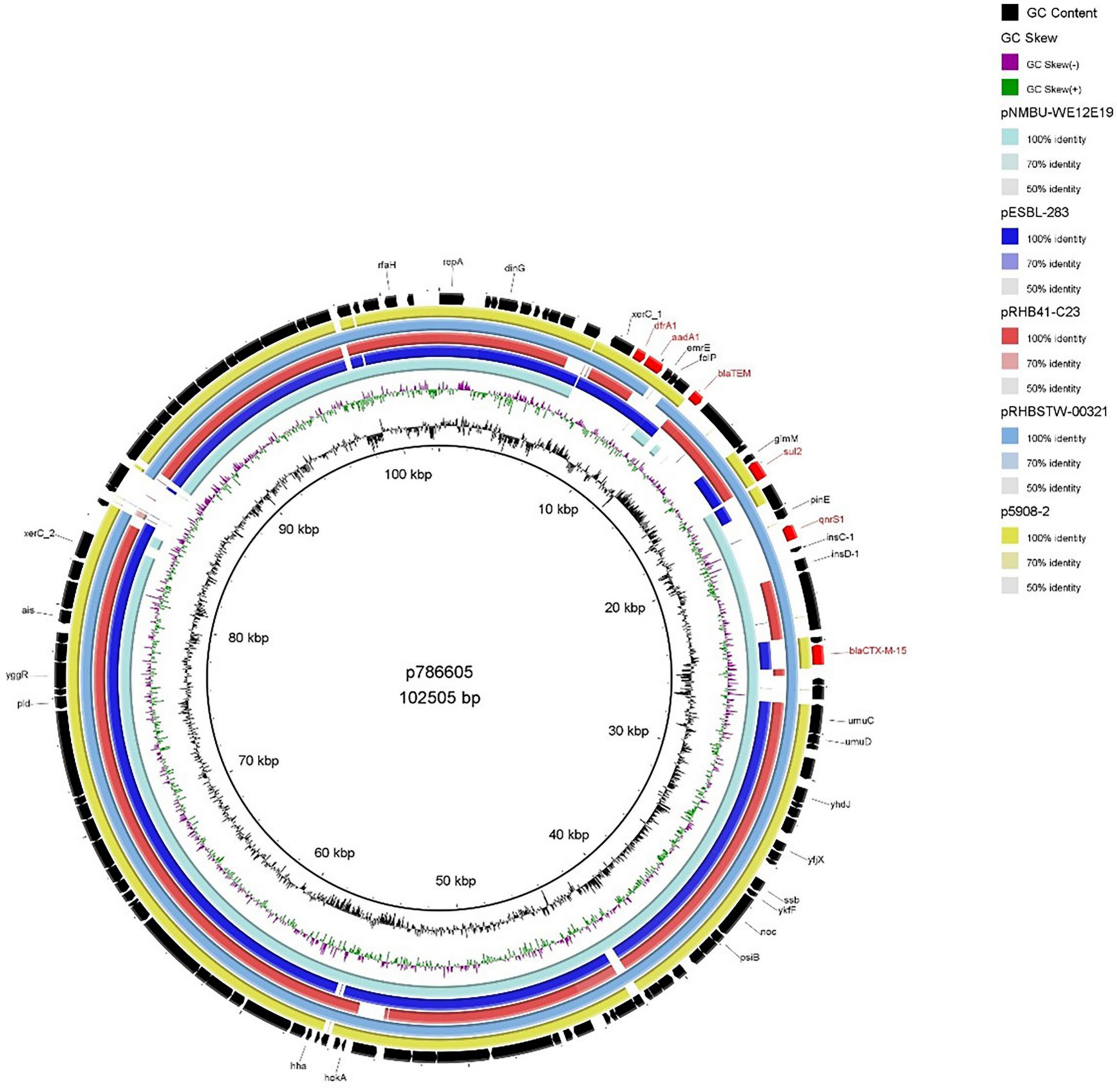
BRIG plot comparing p786605 to five publicly available *bla*_CTX-M-15_ encoding IncI1 plasmids. AMR genes are coloured red, and all other genes are coloured black. Coloured circles represent isolates with a gradient on similarity.

When we compared p786605 to publicly available plasmids (Figure 4), it displayed high conservation in the backbone (the region surrounding the truncated C1 integron and additional AMR determinants) which has been reported previously (Brouwer et al., 2014). While the majority of isolates conveyed sequence similarity to the truncated C1 region only, pRHBSTW-00321 (accession number: CP056606) displayed sequence similarity to the entire region including *sul2*, *qnrS1* and *bla*_CTX-M-15_ AMR determinants.

The co-location of *bla*_CTX-M-15_ with other AMR determinants such as plasmid-mediated quinolone (PMQR) & aminoglycoside resistance genes has been previously reported (Carattoli, 2009; Agyekum et al., 2016; Irrgang et al., 2017; Minja et al., 2021) with further correlation to narrow-spectrum ß-lactamases, sulfonamide & dihydrofolate reductase resistance genes (Singh et al., 2018; Awosile and Agbaje, 2021). These reports are consistent with our findings that *bla*_CTX-M-15_ was associated with PMQR (*qnrS1*), aminoglycoside (*aadA1*), sulfonamide (*sul2*), dihydrofolate reductase (*dfrA1*) and other ß-lactamase resistance (*bla*_TEM_; Figure 4). It has been suggested by Cantón et al. (2012) that these additional AMR genes may maintain *bla*_CTX-M-15_ *via* co-selection processes.

#### p623213

**Figure 5:**
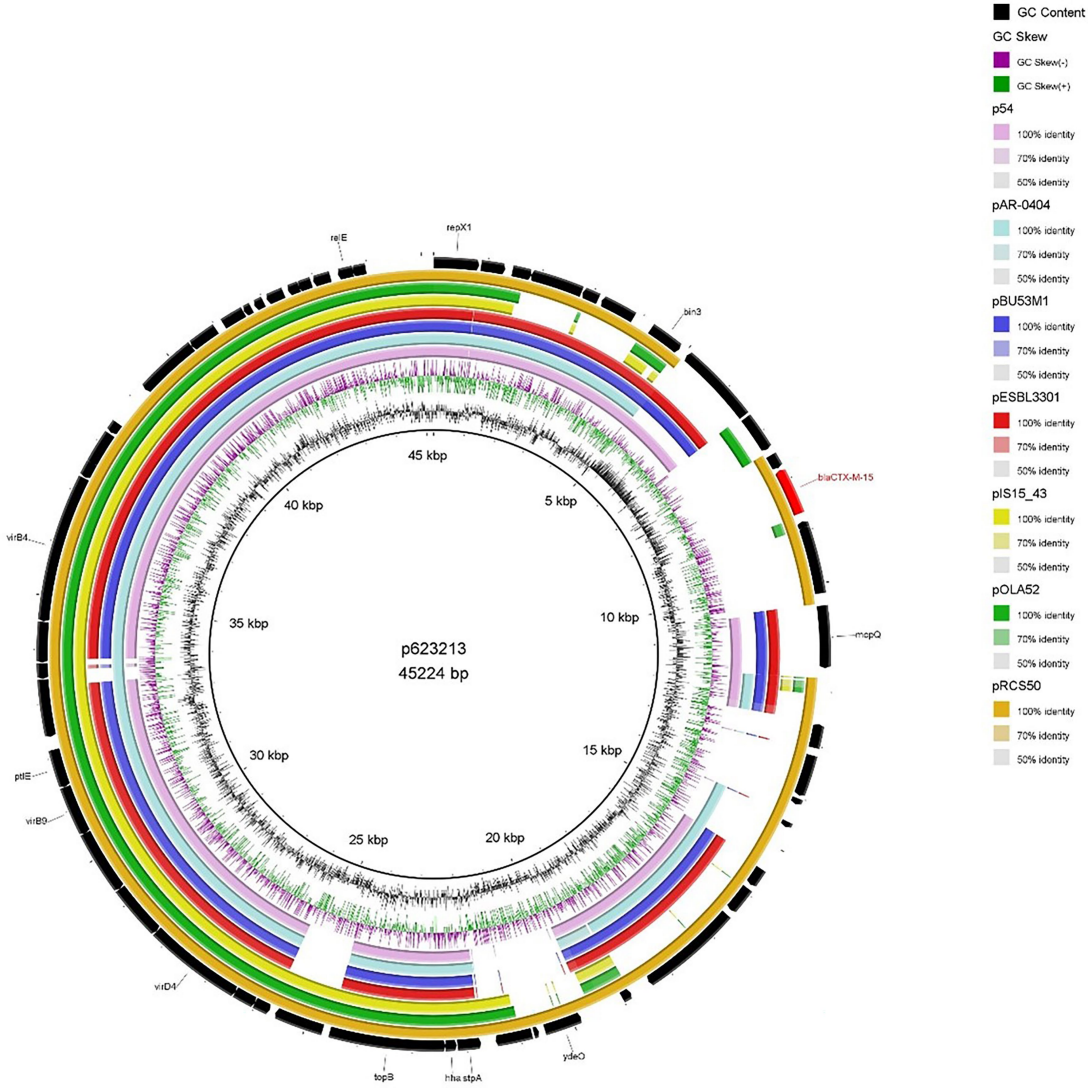
BRIG plot comparing p623213 to seven publicly available *bla*_CTX-M-15_ encoding IncX1 plasmids. AMR genes are coloured red, and all other genes are coloured black. Coloured circles represent isolates with a gradient on similarity.

As mentioned earlier 623213 harboured an IncX1 *bla*_CTX-M-15_–encoding plasmid which was the smallest *bla*_CTX-M-15_ plasmid (46536 bp) detected in our study which is consistent with the report from Branger et al. (2018). When compared to publicly available IncX1 *bla*_CTX-M-15_-encoding plasmid (Figure 5) a high degree of backbone similarity was identified but only pRCS50 (accession number: LT985261.1) showed high sequence similarity (99.93% identity and 91% query coverage), including the region encoding *bla*_CTX-M-15_. Whilst IncX plasmids are associated with a broad range of antibiotics they have a relatively narrow host range (limited to *Enterobacteriaceae*) which could explain why IncX1 plasmids encoding *bla*_CTX-M-15_ are comparatively lesser reported in the literature (Shin and Ko, 2015).

### *bla*_CTX-M-15_ Chromosomal Integrations

The *bla*_CTX-M-15_ identified in 4 of our 8 isolates (542093, 542099, 623214 & 899091) were located on the chromosome, and all were flanked by *ISEcp1*. While other *bla*_CTX-M-15_ insertion sequences have been previously identified, e.g., those involving *ISCR1* and *IS26* (Awosile and Agbaje, 2021; Shawa et al., 2021), only *ISEcp1*-*bla*_CTX-M-15_ was identified in this study but is consistent with literature that it contributes to dissemination and mobilisation of *bla*_CTX-M-15_.

**Figure 6:**
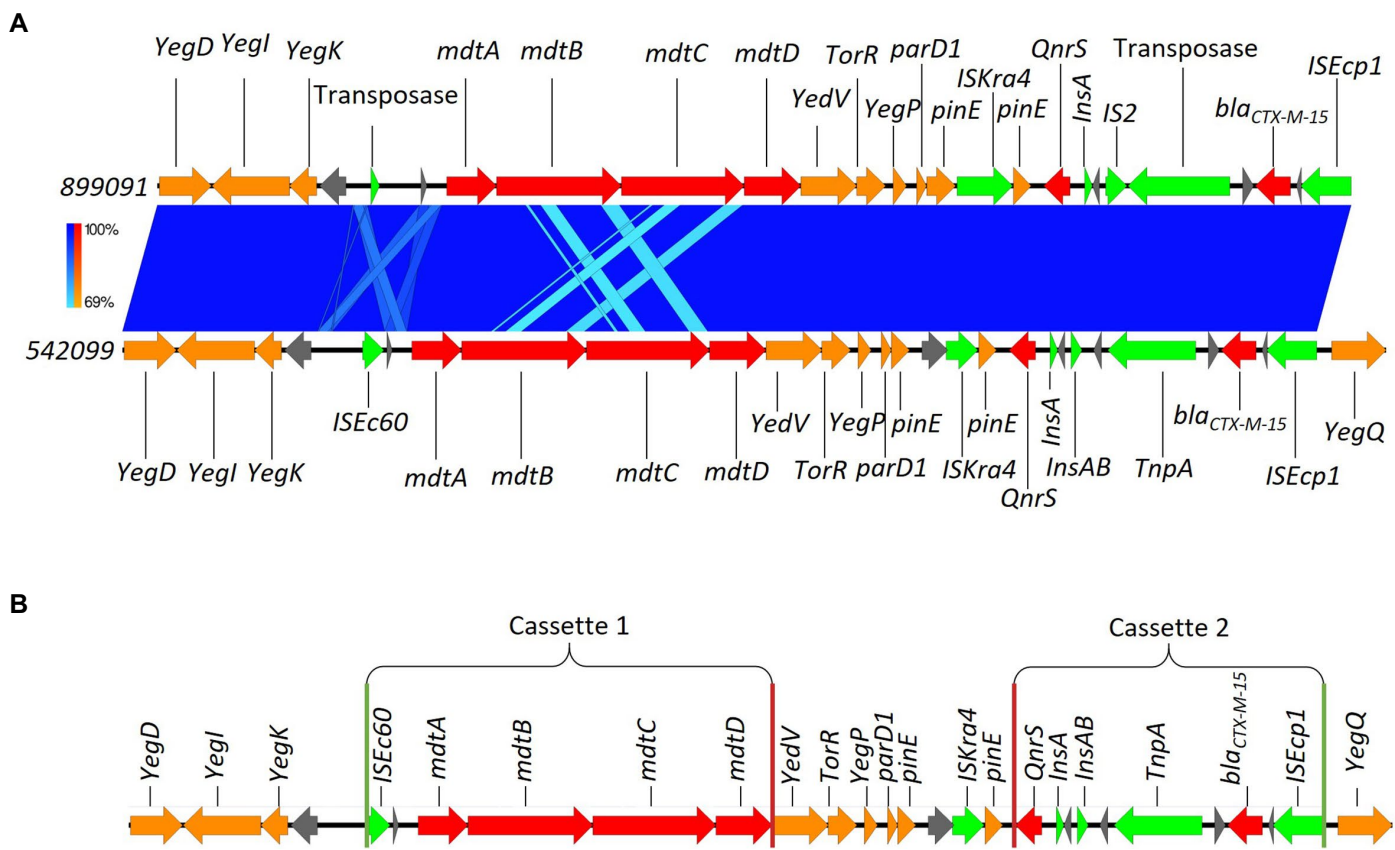
**(A)** Chromosomal integration of *bla*_CTX-M-15_ comparison between isolates 899091 and 542099. Arrows indicate gene direction while colours indicate gene function. Hypothetical proteins are shown in grey; AMR determinants are shown in red; mobile elements are shown in green; and other genes are shown in orange. Scale bars indicate level of sequence similarity for forward (blue). **(B)** Highlights the start and end of AMR cassette regions. Green vertical lines indicate the start of AMR cassettes while red lines indicate the end.

**Figure 7:**
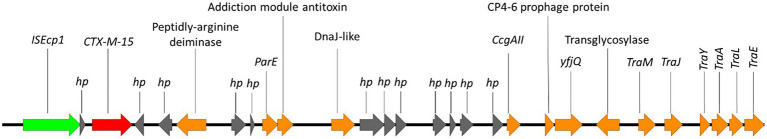
Chromosomal integration of *bla*_CTX-M-15_ in 542093. Arrows indicate gene direction while colours indicate gene function. Hypothetical proteins are shown in grey; AMR determinants are shown in red; mobile elements are shown in green; and other genes are shown in orange.

**Figure 8:**
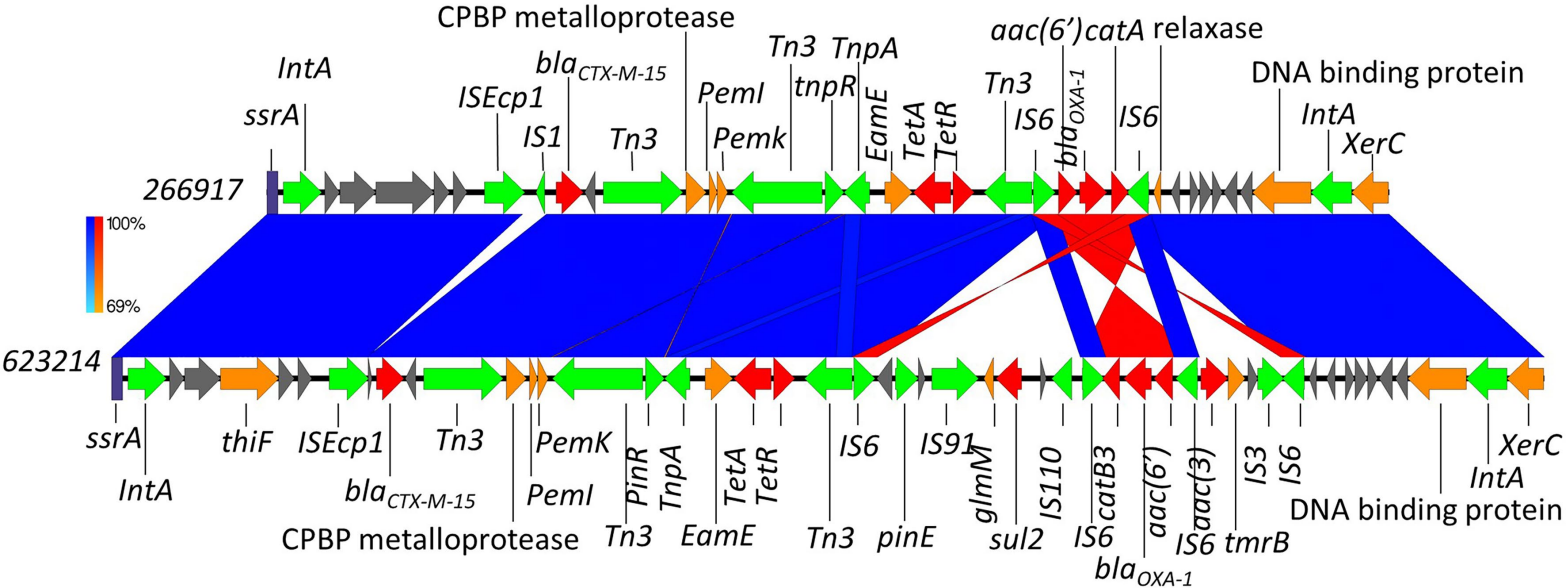
Chromosomal integration of *bla*_CTX-M-15_ comparison between isolates 623214 and 266917. Arrows indicate gene direction while colours indicate gene function. Hypothetical proteins are shown in grey; AMR determinants are shown in red; mobile elements are shown in green; and other genes are shown in orange. Scale bars indicate level of sequence similarity for forward (blue) and reverse (red) sequences.

Here, we identified two isolates (899091 & 542099) that shared the same integration site and also shared the same *bla*_CTX-M-15_-resistant cassette (8,363 bp) containing several multidrug efflux MFS transporters (*mdtA*, *mdtB*, *mdtC* & *mdtD*) and plasmid-mediated quinolone resistance (*qnrS1*; Figure 6). We also identified *ISEcp1*-*bla*_CTX-M-15_ cassette (3,262 bp) integration into a site with plasmid/phage remanence (that was not collated with other resistant determinants) in isolate 542093 (Figure 7). Finally, we identified similarity between the *ISEcp1*-*bla*_CTX-M-15_ cassette integration of isolate 623214 and the publicly available isolate 266917 (accession number: CP026723), where the cassette had integrated into a larger resistance island containing several other AMR determinants (*tetA*, *tetR*, *sul2*, *catB3*, *aac(6′)*, *aac(3)* and *bla*_OXA-1_) and was approximately 65kbp (Figure 8).

#### 899091 and 542099

As mentioned, isolates 899091 & 542099 *bla*_CTX-M-15_ cassette shared a high degree of similarity (99.94% identity; Figure 6) and were also found to integrate into the same chromosomal region despite being isolated from different patients (A & D, respectively) which also had different pathotypes, serotypes and ST. BLAST analysis revealed several samples with a high percent identity with both 899091 & 542099 *bla*_CTX-M-15_ cassette (Supplementary Table 6) indicating that this is not a unique integration of *bla*_CTX-M-15_.

#### 542093

The *bla*_CTX-M-15_ cassette in isolate 542093 integrated into the chromosome at a site with plasmid/phage remanence (Figure 7). Integration of this *bla*_CTX-M-15_ cassette (*ISEcp1* to *TraE*) splits a hypothetical protein and a DUF945 domain-containing protein and contained a myriad of plasmid genes. BLAST analysis of isolates 542093 *bla*_CTX-M-15_ cassette returned no complete hits indicating that this is a unique chromosomal integration of *bla*_CTX-M-15_.

#### 623214

The integration of *bla*_CTX-M-15_ in isolate 623214 was found to be extremely similar to a previously described integration by Greig et al. (2018; accession number: CP026723). Both had integrated into a common phage site between a transfer messenger RNA gene (*ssrA*) and a tyrosine recombinase (*xerC*; Figure 8). The integration also contained several other AMR determinants demonstrating this chromosomal region as a hotspot for integration. The start and end points of these different AMR cassettes have visualised on Figure 8. When compared to publicly available sequences the aforementioned isolate from Greig et al. (2018) was found to be the best alignment (96% query coverage and 99.93% identity). However, three *E. coli* plasmid isolates were also identified to share some nucleotide similarity to isolate 623214–pCS59 (accession number: LT985271), pCS102 (accession number: LT985213) and pRCS22 (accession number: LT985221). These plasmids were found to carry a very similar resistant island to those identified in 623214 but lacked *sul2*.

## Discussion

Polymicrobial infections are often detected in patients reporting travellers’ diarrhoea (Agyekum et al., 2016; Bevan et al., 2018) and maybe caused by multiple exposures to contaminated food or water during the period of travel. A one-off exposure to gross faecal contamination of a single source can also result in the detection of multiple gastrointestinal pathogens from the same patient.

STEC O117:H7 is well established as a cause of travellers’ diarrhoea in travellers returning to the United Kingdom from high-risk regions and is associated with causing persistent gastrointestinal symptoms that can last for weeks or even months (Dallman et al., 2013). On-going sexual transmission among men who have sex with men associated with this serotype has also been described (Baker et al., 2018). The MDR nature of STEC O117:H7 observed in this study has been described previously, although United Kingdom isolates belonging to this serotype did not harbour *bla*_CTX-M-15_. EIEC O96:H19 has been described previously as a cause of foodborne outbreaks of severe gastrointestinal disease in Italy and the UK, most likely linked to asymptomatic food handlers recently returning from travelling to high-risk regions (Newitt et al., 2016). As with STEC O117:H7, previous isolates associated with on-going transmission in the United Kingdome did not carry *bla*_CTX-M-15._

Several ST groups (ST315, ST393, ST405, ST648 and in particular ST131) have previously been described as ‘pandemic clones’ which have significantly contributed to the dissemination of ESBL resistance in both *E. coli* strains as well as other Enterobacteriaceae (Carattoli, 2009; Cantón et al., 2012; Agyekum et al., 2016; Branger et al., 2018; Goswami et al., 2020; Shawa et al., 2021). Moreover, ST groups linked with pandemic clones are usually associated with non-DEC groups as seen with ST131 being associated with UPEC/ExPEC, whereas all isolates in this study were from DEC groups (Forde et al., 2019; Kondratyeva et al., 2020). Here, we described four *bla*_CTX-M-15_-encoding plasmids which were identified from isolates 899037, 786605, 788309 & 623213. Of these four plasmids, we identified one IncI1, one IncX1 and two IncFIB. Furthermore, patient B was infected by two *E. coli* isolates containing two plasmids encoding *bla*_CTX-M-15_ of which both had different incompatibility types (IncI1 & IncFIB, respectively); therefore, plasmid transmission from one strain to the other in the gut was unlikely. Both Patients A & C only contained one plasmid encoding *bla*_CTX-M-15_ isolates (899037 & 623213, respectively) on IncFIB and IncX1 plasmids, respectively. It has been shown previously that *bla*_CTX-M-15_ is mainly associated with multi-replicon IncF (IncFIA, IncFIB and IncFII) plasmids, which is consistent with the results of this study despite being a small cohort of isolates (Cantón et al., 2012; Agyekum et al., 2016; Irrgang et al., 2017; Branger et al., 2018). Whilst a broad range of other replicon plasmids (including IncI) have been associated with the dissemination of *bla*_CTX-M-15_, IncX on the other hand, hasn’t been reported with a high incidence of carriage of *bla*_CTX-M-15_ in DEC isolates (Branger et al., 2018).

Other identical plasmids encoding *bla*_CTX-M-15_ have been identified across genetically diverse DEC strains and were termed as ‘epidemic plasmids’ (e.g., pC151a, pEK516; Lavollay et al., 2006; Carattoli, 2009; Bevan et al., 2021). Further investigations are required to confirm if p788309 & p899037 are ‘epidemic plasmids’ and it would be interesting to monitor the literature to see if any more similar plasmids are identified.

A plethora of literature has described the genetic environment of *bla*_CTX-M-15_ (Huang et al., 2017; Singh et al., 2018; Yoon et al., 2020; Awosile and Agbaje, 2021; Shawa et al., 2021); recently, Nair et al. (2021) described how *bla*_CTX-M-15_ can easily transpose into plasmids as they are flanked by ISEc9/ISEc1 and another IS/*tnpA* element that may facilitate translocation. One of the objectives of the current study was to investigate the mechanism involved in the transmission of genes (*bla*_CTX-M_) involved in resistance to ESBL, such as mobile genetic elements present either on plasmids or the chromosome. Understanding these mechanisms are critical in elucidating the possibility of how other resistant genes in particular carbapenem resistance genes can be transmitted, as carbapenem resistance is a major problem globally and in particular in Pakistan (Bilal et al., 2021; Hadjadj et al., 2021). Furthermore, accurately characterising plasmids about AMR genes and other virulence factors is crucial in limiting as well as understanding the dissemination of AMR (Berbers et al., 2020). Even though plasmids encoding AMR genes will place a selective burden on the host cells (due to replication and translation of plasmid genes), antibiotic usage provides a selective pressure for plasmid maintenance (Goswami et al., 2020).

C1 integrons are known to easily disseminate AMR determinants throughout a bacterial population but despite this, they are still poorly understood (Ghaly et al., 2017; Singh et al., 2018; Kubomura et al., 2020). Recently, C1 integrons were found to be commonly associated, often located adjacent to IS elements, such as *ISEcp1* responsible for mobilisation of *bla*_CTX-M-15_ (Awosile and Agbaje, 2021). This close association is consistent with our findings regarding a truncated C1 integron in p786605. It is likely that these C1 integrons contribute to the persistence and dissemination of resistant determinants (most importantly *bla*_CTX-M-15_) under selective pressures of other commonly used antibiotics and should therefore be monitored closely (Jiang et al., 2019).

Chromosomal integration was first reported by Chanawong et al. (2002) and was reported as an uncommon event when compared to plasmid-encoded *bla*_CTX-M-15_ (Harada et al., 2012). However, reports of chromosomally encoded *bla*_CTX-M-15_ have increased in recent years (Agyekum et al., 2016; Huang et al., 2017; Irrgang et al., 2017; Decano et al., 2019; Ludden et al., 2020; Yoon et al., 2020; Bevan et al., 2021; Shawa et al., 2021) suggesting it is no longer considered a rare event. Decano et al. (2019) recently identified three identical chromosomally located *bla*_CTX-M-15_ that were not tandem repeats all mediated by *ISEcp1* (at the 5′ end) which is consistent with the findings of this study and demonstrate the increased identification of *bla*_CTX-M-15_ chromosomal integration. The increase in chromosomal integrations could be due to the increase in antibiotic selective pressures. Hence, investigating the propensity of chromosomal integration of *bla*_CTX-M-15_ over the last 10 years and to see if there has been an increase in the number of isolates with chromosomally located *bla*_CTX-M-15_ will be/should be a critical undertaking for the future.

BLAST analysis was completed to understand if the chromosomal integrations identified in this study were unique or previously seen, therefore contributing to the suggestion that chromosomal integrations are becoming more common. BLAST analysis released that chromosomal integration of *bla*_CTX-M-15_ for isolate 542093 was unique due to no complete hits being returned. However, chromosomal integration for the near identical *bla*_CTX-M-15_ cassettes for isolates 899091 & 542099 did return several significant near complete hits (supplementary Table 6), which indicates that this chromosomal integration is not unique and would explain why it was identified in both 899091 & 542099. Finally, 623214 was found to share significant nucleotide similarity to a previously published chromosomal integration of *bla*_CTX-M-15_ and is therefore not unique but 623214 had acquired addition AMR determinants (Figure 8) compared to 266917 from Greig et al. (2018).

The variety of chromosomal integration sites for the *ISEcp1*-*bla*_CTX-M-15_ cassette is most likely mediated by the IS promoter upstream of *bla*_CTX-M-15_. While all chromosomal integrations in this study shared the same IS promoter upstream of *bla*_CTX-M-15_ (*ISEcp1*) we still identified different chromosomal integration sites which would suggest other mechanisms are contributing to *bla*_CTX-M-15_ chromosomal integration. A potential contributing factor could be the resistant cassettes’ genetic environment, as we identified shared cassette environment and integration site between isolates 899091 & 542099 (Figure 6) as well as 623214 & 266917 (accession number: CP026723; Figure 8). Understanding the importance of that AMR-based MGE integration into the chromosome could be crucial for curbing the dissemination of ESBL resistance.

Chromosomal integration events are believed to occur when the cost of maintaining plasmids (encoding AMR genes such as *bla*_CTX-M-15_) outweighs the benefits of the AMR cassette. This cost of maintenance will then selectively put pressure on the MGE (containing the AMR gene) to integrate into the chromosome thus reducing the cost but keeping the AMR benefit. This would also explain the existence of megaplasmids—why maintain two different plasmids when you can maintain a larger plasmid at a lower cost. However, while it has been described that ESBL plasmids confer a fitness cost to the host strain (Andersson and Hughes, 2010) it has also been shown that there is comparatively little to any cost (Melnyk et al., 2015; Vogwill and Maclean, 2015). Unfortunately, neither the benefits nor the costs of chromosomal integration of *bla*_CTX-M-15_ are fully understood, but here, we have shown that integration events are occurring, and the stable maintenance of crucial AMR genes seems to be a viable safeguard for survival and continued AMR spread. This is supported by our observation of isolates 542099, 623214 & 899091 which despite carrying an accessory plasmid did not encode any AMR genes outside of the ones encoded chromosomally.

## Conclusion

The initial aim of this study was to describe an end-to-end protocol for the routine analysis of plasmids in GI pathogens using long-read sequencing. However, we found that in half of the isolates in this small dataset, *bla*_CTX-M-15_ had been incorporated into the chromosome at three different sites, and so we also described our protocol for analysing AMR gene insertion sites.

The plasmids exhibited three different replicon types in three different DEC pathotypes, and there was no evidence that the *bla*_CTX-M-15_ plasmids had been exchanged *in vivo*. Characterising and understanding *bla*_CTX-M-15_-encoding plasmids as well as chromosomal integration events will inform strategies to ease the burden and spread of AMR.

Determining the mechanisms that contribute to the global spread of AMR will confer improvements in infection prevention and allow for the conservation of existing antibiotics. We have confirmed both the importance of plasmids and chromosomal integration of the *bla*_CTX-M-15_ enzyme and commented on the importance travellers play in the dissemination of ESBL resistance.

There is evidence that travellers returning to the United Kingdome from high-risk regions are inadvertently importing MDR bacteria into their gut. Characterisation of these bacteria and the AMR determinants and mobile genetic elements they carry is essential to better understand their source, mechanisms of persistence and transmission, and to ultimately reduce the threat to public health.

## Data Availability Statement

The datasets presented in this study can be found in online repositories. The names of the repository/repositories and accession number(s) can be found in the article/Supplementary Material.

## Author Contributions

DRG completed the Nanopore sequencing using ONT, data processing, *de novo* assembly and polishing. The short-read sequencing on the Illumina HiSeq 2500 was performed as part of the routine service for the UKHSA. DRG, SN, and CJ designed the project and helped to revised the manuscript. MTB completed comparative genomic analysis and figure generation. All authors contributed to the article and approved the submitted version.

## Funding

Health Protection Research Unit (HPRU) in Genomics and Enabling Data is a collaboration funded by the National Institute for Health Research (NIHR) between UK Health Security Agency (UKHSA), the University of Warwick, the Centre for Genomic Pathogen Surveillance and the University of Cambridge. Health Protection Research Unit (HPRU) in Gastrointestinal Infections is a collaboration funded by the National Institute for Health Research (NIHR) at the University of Liverpool in partnership with UK Health Security Agency (UKHSA) and the University of Warwick.

## Conflict of Interest

The authors declare that the research was conducted in the absence of any commercial or financial relationships that could be construed as a potential conflict of interest.

## Publisher’s Note

All claims expressed in this article are solely those of the authors and do not necessarily represent those of their affiliated organizations, or those of the publisher, the editors and the reviewers. Any product that may be evaluated in this article, or claim that may be made by its manufacturer, is not guaranteed or endorsed by the publisher.

## Footnotes

^1^ https://github.com/rrwick/Porechop

^2^ https://github.com/rrwick/Filtlong

^3^ https://cge.cbs.dtu.dk/services/PlasmidFinder/

## References

[ref1] Achtman M. Wain J. Weill F. X. Nair S. Zhou Z. Sangal V.. (2012). Multilocus sequence typing as a replacement for serotyping in salmonella enterica. PLoS Pathog. 8:e1002776. doi: 10.1371/journal.ppat.1002776, PMID: 22737074 PMC3380943

[ref2] Agyekum A. Fajardo-Lubián A. Ansong D. Partridge S. R. Agbenyega T. Iredell J. R. (2016). blaCTX-M-15 carried by IncF-type plasmids is the dominant ESBL gene in *Escherichia coli* and Klebsiella pneumoniae at a hospital in Ghana. Diagn. Microbiol. Infect. Dis. 84, 328–333. doi: 10.1016/j.diagmicrobio.2015.12.010, PMID: 26830052

[ref3] Alikhan N. F. Petty N. K. Ben Zakour N. L. Beatson S. A. (2011). BLAST ring image generator (BRIG): simple prokaryote genome comparisons. BMC Genomics 12, 1–10. doi: 10.1186/1471-2164-12-402, PMID: 21824423 PMC3163573

[ref4] Andersson D. I. Hughes D. (2010). Antibiotic resistance and its cost: is it possible to reverse resistance? Nat. Rev. Microbiol. 8, 260–271. doi: 10.1038/nrmicro2319, PMID: 20208551

[ref5] Awosile B. B. Agbaje M. (2021). Genetic environments of plasmid-mediated blaCTXM-15 Beta-lactamase gene in Enterobacteriaceae from Africa. Microbiol. Res. 12, 383–394. doi: 10.3390/microbiolres12020026

[ref6] Baker K. S. Dallman T. J. Thomson N. R. Jenkins C. (2018). An outbreak of a rare Shiga-toxin-producing *Escherichia coli* serotype (O117:H7) among men who have sex with men. Microb. Genom. 4:e000181. doi: 10.1099/mgen.0.000181, PMID: 29781799 PMC6113874

[ref7] Berbers B. Saltykova A. Garcia-Graells C. Philipp P. Arella F. Marchal K.. (2020). Combining short and long read sequencing to characterize antimicrobial resistance genes on plasmids applied to an unauthorized genetically modified bacillus. Sci. Rep. 10, 4310–4313. doi: 10.1038/s41598-020-61158-0, PMID: 32152350 PMC7062872

[ref8] Bevan E. R. McNally A. Thomas C. M. Piddock L. J. V. Hawkey P. M. (2018). Acquisition and loss of CTX-M-producing and non-producing *Escherichia coli* in the fecal microbiome of travelers to South Asia. mBio 9:e2408-18. doi: 10.1128/mBio.02408-18, PMID: 30538187 PMC6299485

[ref9] Bevan E. R. Powell M. J. Toleman M. A. Thomas C. M. Piddock L. J. V. Hawkey P. M. (2021). Molecular characterization of plasmids encoding blaCTX-M from faecal *Escherichia coli* in travellers returning to the UK from South Asia. J. Hosp. Infect. 114, 134–143. doi: 10.1016/j.jhin.2021.03.030 33862156

[ref10] Bilal H. Khan M. N. Rehman T. Hameed M. F. Yang X. (2021). Antibiotic resistance in Pakistan: a systematic review of past decade. BMC Infect. Dis. 21, 244–219. doi: 10.1186/s12879-021-05906-1, PMID: 33676421 PMC7937258

[ref11] Bolger A. M. Lohse M. Usadel B. (2014). Trimmomatic: a flexible trimmer for Illumina sequence data. Bioinformatics 30, 2114–2120. doi: 10.1093/bioinformatics/btu170, PMID: 24695404 PMC4103590

[ref12] Boxall M. D. Day M. R. Greig D. R. Jenkins C. (2020). Antimicrobial resistance profiles of diarrhoeagenic *Escherichia coli* isolated from travellers returning to the UK, 2015-2017. J. Med. Microbiol. 69, 932–943. doi: 10.1099/JMM.0.001214, PMID: 32530393

[ref13] Branger C. Ledda A. Billard-Pomares T. Doublet B. Fouteau S. Barbe V.. (2018). Extended-spectrum β-lactamase-encoding genes are spreading on a wide range of *Escherichia coli* plasmids existing prior to the use of third-generation cephalosporins. Microb. Genom. 4:203. doi: 10.1099/mgen.0.000203, PMID: 30080134 PMC6202452

[ref14] Brouwer M. S. M. Bossers A. Harders F. van Essen-Zandbergen A. Mevius D. J. Smith H. E. (2014). Complete genome sequences of IncI1 plasmids carrying extendedspectrum β-lactamase genes. Genome Announc. 2, 859–873. doi: 10.1128/genomeA.00859-14, PMID: 25169863 PMC4148731

[ref15] Cantón R. González-Alba J. M. Galán J. C. (2012). CTX-M enzymes: origin and diffusion. Front. Microbiol. 3:110. doi: 10.3389/fmicb.2012.00110, PMID: 22485109 PMC3316993

[ref16] Carattoli A. (2009). Resistance plasmid families in Enterobacteriaceae. Antimicrob. Agents Chemother. 53, 2227–2238. doi: 10.1128/AAC.01707-08, PMID: 19307361 PMC2687249

[ref17] Carver T. Harris S. R. Berriman M. Parkhill J. McQuillan J. A. (2012). Artemis: an integrated platform for visualization and analysis of high-throughput sequence-based experimental data. Bioinformatics 28, 464–469. doi: 10.1093/bioinformatics/btr703, PMID: 22199388 PMC3278759

[ref18] Casburn-Jones A. C. Farthing M. J. G. (2004). Management of infectious diarrhoea. Manage. infect. Diarrhoea. Gut 53, 296–305. doi: 10.1136/gut.2003.022103, PMID: 14724167 PMC1774945

[ref19] Chanawong A. M’Zali F. H. Heritage J. Xiong J. H. Hawkey P. M. (2002). Three cefotaximases, CTX-M-9, CTX-M-13, and CTX-M-14, among Enterobacteriaceae in the People’s republic of China. Antimicrob. Agents Chemother. 46, 630–637. doi: 10.1128/AAC.46.3.630-637.2002, PMID: 11850241 PMC127467

[ref20] Chattaway M. A. Schaefer U. Tewolde R. Dallman T. J. Jenkins C. (2017). Identification of *Escherichia coli* and shigella species from whole-genome sequences. J. Clin. Microbiol. 55, 616–623. doi: 10.1128/JCM.01790-16, PMID: 27974538 PMC5277532

[ref21] Dallman T. Cross L. Bishop C. Perry N. Olesen B. Grant K. A.. (2013). Whole genome sequencing of an unusual serotype of Shiga toxin-producing *Escherichia coli*. Emerg. Infect. Dis. 19, 1302–1304. doi: 10.3201/eid1908.130016, PMID: 23877005 PMC3739510

[ref22] Darling A. C. E. Mau B. Blattner F. R. Perna N. T. (2004). Mauve: multiple alignment of conserved genomic sequence with rearrangements. Genome Res. 14, 1394–1403. doi: 10.1101/gr.2289704, PMID: 15231754 PMC442156

[ref23] Decano A. G. Ludden C. Feltwell T. Judge K. Parkhill J. Downing T. (2019). Complete assembly of *Escherichia coli* sequence type 131 genomes using long reads demonstrates antibiotic resistance gene variation within diverse plasmid and chromosomal contexts. mSphere 4:19. doi: 10.1128/msphere.00130-19 PMC6506616 31068432

[ref24] Do Nascimento V. Day M. R. Doumith M. Hopkins K. L. Woodford N. Godbole G.. (2017). Comparison of phenotypic and WGS-derived antimicrobial resistance profiles of enteroaggregative *Escherichia coli* isolated from cases of diarrhoeal disease in England, 2015-16. J. Antimicrob. Chemother. 72, 3288–3297. doi: 10.1093/jac/dkx301, PMID: 28961934

[ref25] Feldgarden M. Brover V. Gonzalez-Escalona N. Frye J. G. Haendiges J. Haft D. H.. (2021). AMRFinderPlus and the reference gene catalog facilitate examination of the genomic links among antimicrobial resistance, stress response, and virulence. Sci. Rep. 11:12728. doi: 10.1038/s41598-021-91456-0, PMID: 34135355 PMC8208984

[ref26] Forde B. M. Roberts L. W. Phan M. D. Peters K. M. Fleming B. A. Russell C. W.. (2019). Population dynamics of an *Escherichia coli* ST131 lineage during recurrent urinary tract infection. Nat. Commun. 10, 3643–3610. doi: 10.1038/s41467-019-11571-5, PMID: 31409795 PMC6692316

[ref27] Galata V. Fehlmann T. Backes C. Keller A. (2019). PLSDB: a resource of complete bacterial plasmids. Nucleic Acids Res. 47, D195–D202. doi: 10.1093/nar/gky1050, PMID: 30380090 PMC6323999

[ref28] Gekenidis M. T. Rigotti S. Hummerjohann J. Walsh F. Drissner D. (2020). Long-term persistence of blactx-m-15 in soil and lettuce after introducing extended-spectrum β-lactamase (Esbl)-producing *Escherichia coli via* manure or water. Microorganisms 8, 1–18. doi: 10.3390/microorganisms8111646, PMID: 33114244 PMC7690902

[ref29] Ghaly T. M. Chow L. Asher A. J. Waldron L. S. Gillings M. R. (2017). Evolution of class 1 integrons: mobilization and dispersal via food-borne bacteria. PLoS One 12:e0179169. doi: 10.1371/journal.pone.0179169, PMID: 28586403 PMC5460862

[ref30] Gomes T. A. T. Elias W. P. Scaletsky I. C. A. Guth B. E. C. Rodrigues J. F. Piazza R. M. F.. (2016). Diarrheagenic *Escherichia coli*. Brazilian. J. Microbiol. 47(Suppl 1), 3–30. doi: 10.1016/j.bjm.2016.10.015, PMID: 27866935 PMC5156508

[ref31] Goswami C. Fox S. Holden M. T. G. Connor M. Leanord A. Evans T. J. (2020). Origin, maintenance and spread of antibiotic resistance genes within plasmids and chromosomes of bloodstream isolates of *Escherichia coli*. Microb. Genom. 6:353. doi: 10.1099/mgen.0.000353, PMID: 32160146 PMC7276700

[ref32] Greig D. R. Dallman T. J. Hopkins K. L. Jenkins C. (2018). MinION nanopore sequencing identifies the position and structure of bacterial antibiotic resistance determinants in a multidrug-resistant strain of enteroaggregative *Escherichia coli*. Microb. Genom. 4:213. doi: 10.1099/MGEN.0.000213, PMID: 30235111 PMC6249433

[ref33] Greig D. R. Jenkins C. Gharbia S. E. Dallman T. J. (2021). Analysis of a small outbreak of Shiga toxin-producing *Escherichia coli* O157: H7 using long-read sequencing. Microb. Genom. 7:545. doi: 10.1099/mgen.0.000545, PMID: 33683192 PMC8190617

[ref34] Guarino A. Bruzzese E. Giannattasio A. (2018). Antibiotic treatment of acute gastroenteritis in children. F1000 Res. 7:193. doi: 10.12688/f1000research.12328.1, PMID: 29511533 PMC5814741

[ref35] Hadjadj L. Syed M. A. Abbasi S. A. Rolain J. M. Jamil B. (2021). Diversity of Carbapenem resistance mechanisms in clinical gram-negative bacteria in Pakistan. Microb. Drug Resist. 27, 760–767. doi: 10.1089/mdr.2019.0387, PMID: 33211640

[ref36] Harada S. Ishii Y. Saga T. Kouyama Y. Tateda K. Yamaguchi K. (2012). Chromosomal integration and location on IncT plasmids of the Bla CTX-M-2 gene in Proteus mirabilis clinical isolates. Antimicrob. Agents Chemother. 56, 1093–1096. doi: 10.1128/AAC.00258-11, PMID: 22106217 PMC3264238

[ref37] Huang W. Wang G. Sebra R. Zhuge J. Yin C. Aguero-Rosenfeld M. E.. (2017). Emergence and evolution of multidrug-resistant Klebsiella pneumoniae with both blaKPC and blaCTX-M integrated in the chromosome. Antimicrob. Agents Chemother. 61:17. doi: 10.1128/AAC.00076-17, PMID: 28438939 PMC5487645

[ref38] Irrgang A. Falgenhauer L. Fischer J. Ghosh H. Guiral E. Guerra B.. (2017). CTX-M-15-producing *E. coli* isolates from food products in Germany are mainly associated with an IncF-type plasmid and belong to two predominant clonal *E. coli* lineages. Front. Microbiol. 8:2318. doi: 10.3389/fmicb.2017.02318, PMID: 29209306 PMC5702323

[ref39] Jiang H. Cheng H. Liang Y. Yu S. Yu T. Fang J.. (2019). Diverse Mobile genetic elements and conjugal transferability of sulfonamide resistance genes (sul1, sul2, and sul3) in *Escherichia coli* isolates From Penaeus vannamei and pork From large Markets in Zhejiang. Front. Virol. 10:1787. doi: 10.3389/fmicb.2019.01787, PMID: 31428076 PMC6690019

[ref40] Kaper J. B. Nataro J. P. Mobley H. L. T. (2004). Pathogenic *Escherichia coli*. Nat. Rev. Microbiol. 2, 123–140. doi: 10.1038/nrmicro818 15040260

[ref41] Kolmogorov M. Yuan J. Lin Y. Pevzner P. A. (2019). Assembly of long, error-prone reads using repeat graphs. Nat. Biotechnol. 37, 540–546. doi: 10.1038/s41587-019-0072-8, PMID: 30936562

[ref42] Kondratyeva K. Salmon-Divon M. Navon-Venezia S. (2020). Meta-analysis of pandemic *Escherichia coli* ST131 Plasmidome proves restricted plasmid-clade associations. Sci. Rep. 10, 36–11. doi: 10.1038/s41598-019-56763-7, PMID: 31913346 PMC6949217

[ref43] Kubomura A. Sekizuka T. Onozuka D. Murakami K. Kimura H. Sakaguchi M.. (2020). Truncated class 1 Integron gene cassette arrays contribute to antimicrobial resistance of Diarrheagenic *Escherichia coli*. Biomed. Res. Int. 2020, 1–9. doi: 10.1155/2020/4908189, PMID: 32090095 PMC7013361

[ref44] Lavollay M. Mamlouk K. Frank T. Akpabie A. Burghoffer B. Ben Redjeb S.. (2006). Clonal dissemination of a CTX-M-15 β-lactamase-producing *Eschenchia coli* strain in the Paris area, Tunis, and Bangui. Antimicrob. Agents Chemother. 50, 2433–2438. doi: 10.1128/AAC.00150-06, PMID: 16801423 PMC1489776

[ref45] Lee W. W. Y. Mattock J. Greig D. R. Langridge G. C. Baker D. Bloomfield S.. (2021). Characterization of a pESI-like plasmid and analysis of multidrug-resistant salmonella enterica Infantis isolates in England and Wales. Microb. Genom. 7:000658. doi: 10.1099/mgen.0.000658, PMID: 34647862 PMC8627215

[ref46] Loman N. J. Quick J. Simpson J. T. (2015). A complete bacterial genome assembled de novo using only nanopore sequencing data. Nat. Methods 12, 733–735. doi: 10.1038/nmeth.3444, PMID: 26076426

[ref47] Ludden C. Decano A. G. Jamrozy D. Pickard D. Morris D. Parkhill J.. (2020). Genomic surveillance of *Escherichia coli* ST131 identifies local expansion and serial replacement of subclones. Microb. Genom. 6:e000352. doi: 10.1099/mgen.0.000352, PMID: 32213258 PMC7276707

[ref48] Mazumder R. Abdullah A. Ahmed D. Hussain A. (2020). High prevalence of blactx-m-15 gene among extended-spectrum β-lactamase-producing *Escherichia coli* isolates causing extraintestinal infections in Bangladesh. Antibiotics 9, 1–11. doi: 10.3390/antibiotics9110796, PMID: 33187055 PMC7696227

[ref49] Melnyk A. H. Wong A. Kassen R. (2015). The fitness costs of antibiotic resistance mutations. Evol. Appl. 8, 273–283. doi: 10.1111/eva.12196, PMID: 25861385 PMC4380921

[ref50] Minja C. A. Shirima G. Mshana S. E. (2021). Conjugative plasmids disseminating ctx-m-15 among human, animals and the environment in Mwanza Tanzania: a need to intensify one health approach. Antibiotics 10:836. doi: 10.3390/antibiotics10070836, PMID: 34356757 PMC8300620

[ref51] Nair S. Chattaway M. Langridge G. C. Gentle A. Day M. Ainsworth E. V.. (2021). ESBL-producing strains isolated from imported cases of enteric fever in England and Wales reveal multiple chromosomal integrations of blaCTX-M-15in XDR salmonella Typhi. J. Antimicrob. Chemother. 76, 1459–1466. doi: 10.1093/jac/dkab049 33704480

[ref52] Newitt S. MacGregor V. Robbins V. Bayliss L. Anne Chattaway M. Dallman T.. (2016). Two linked enteroinvasive *Escherichia coli* outbreaks, Nottingham, UK, June 2014. Emerg. Infect. Dis. 22, 1178–1184. doi: 10.3201/eid2207.152080, PMID: 27314432 PMC4918187

[ref53] Penders J. Stobberingh E. E. Savelkoul P. H. M. Wolffs P. F. G. (2013). The human microbiome as a reservoir of antimicrobial resistance. Front. Microbiol. 4:87. doi: 10.3389/fmicb.2013.00087, PMID: 23616784 PMC3627978

[ref54] Rédei G. P. (2008). “Blast (basic local alignment search tool),” in Encyclopedia of Genetics, Genomics, Proteomics, and Informatics (United States: Springer Science & Business Media), 221–221.

[ref55] Seemann T. (2014). Prokka: rapid prokaryotic genome annotation. Bioinformatics 30, 2068–2069. doi: 10.1093/bioinformatics/btu153, PMID: 24642063

[ref56] Shaikh S. Fatima J. Shakil S. Rizvi S. M. D. Kamal M. A. (2015). Antibiotic resistance and extended spectrum beta-lactamases: types, epidemiology and treatment. Saudi J. Biol. Sci. 22, 90–101. doi: 10.1016/j.sjbs.2014.08.002, PMID: 25561890 PMC4281622

[ref57] Shawa M. Furuta Y. Mulenga G. Mubanga M. Mulenga E. Zorigt T.. (2021). Novel chromosomal insertions of ISEcp1-Bla CTX-M-15 and diverse antimicrobial resistance genes in Zambian clinical isolates of Enterobacter cloacae and Escherichia coli. Antimicrob. Resist. Infect. Control 10:8, 79. doi: 10.1186/s13756-021-00941-8, PMID: 33971966 PMC8111917

[ref58] Shin J. Ko K. S. (2015). Effect of plasmids harbouring blaCTX-M on the virulence and fitness of Escherichia coli ST131 isolates. Int. J. Antimicrob. Agents 46, 214–218. doi: 10.1016/j.ijantimicag.2015.04.012, PMID: 26116415

[ref59] Singh N. S. Singhal N. Virdi J. S. (2018). Genetic environment of blaTEM-1, blaCTX-M-15, blaCMY-42 and characterization of integrons of Escherichia coli isolated from an Indian urban aquatic environment. Front. Microbiol. 9:382. doi: 10.3389/fmicb.2018.00382, PMID: 29563901 PMC5845874

[ref60] Sullivan M. J. Petty N. K. Beatson S. A. (2011). Easyfig: a genome comparison visualizer. Bioinformatics 27, 1009–1010. doi: 10.1093/bioinformatics/btr039, PMID: 21278367 PMC3065679

[ref61] Tewolde R. Dallman T. Schaefer U. Sheppard C. L. Ashton P. Pichon B.. (2016). MOST: a modified MLST typing tool based on short read sequencing. PeerJ 4:e2308. doi: 10.7717/peerj.2308, PMID: 27602279 PMC4991843

[ref62] Vaser R. Sović I. Nagarajan N. Šikić M. (2017). Fast and accurate de novo genome assembly from long uncorrected reads. Genome Res. 27, 737–746. doi: 10.1101/gr.214270.116, PMID: 28100585 PMC5411768

[ref63] Vogwill T. Maclean R. C. (2015). The genetic basis of the fitness costs of antimicrobial resistance: A meta-analysis approach. Evol. Appl. 8, 284–295. doi: 10.1111/eva.12202, PMID: 25861386 PMC4380922

[ref64] Walker B. J. Abeel T. Shea T. Priest M. Abouelliel A. Sakthikumar S.. (2014). Pilon: an integrated tool for comprehensive microbial variant detection and genome assembly improvement. PLoS One 9:e112963. doi: 10.1371/journal.pone.0112963, PMID: 25409509 PMC4237348

[ref65] Wick R. R. Judd L. M. Gorrie C. L. Holt K. E. (2017). Unicycler: resolving bacterial genome assemblies from short and long sequencing reads. PLoS Comput. Biol. 13:e1005595. doi: 10.1371/journal.pcbi.1005595, PMID: 28594827 PMC5481147

[ref66] Yara D. A. Greig D. R. Gally D. L. Dallman T. J. Jenkins C. (2020). Comparison of Shiga toxin-encoding bacteriophages in highly pathogenic strains of Shiga toxin-producing Escherichia coli O157:H7 in the UK. Microb. Genom. 6:334. doi: 10.1099/mgen.0.000334, PMID: 32100710 PMC7200060

[ref67] Yoon E.-J. Gwon B. Liu C. Kim D. Won D. Park S. G.. (2020). Beneficial Chromosomal Integration of the Genes for CTX-M Extended-Spectrum β-Lactamase in Klebsiella pneumoniae for Stable Propagation. mSystems 5:e00459-20. doi: 10.1128/msystems.00459-20 32994286 PMC7527135

